# Macrophages Are Required for Dendritic Cell Uptake of Respiratory Syncytial Virus from an Infected Epithelium

**DOI:** 10.1371/journal.pone.0091855

**Published:** 2014-03-20

**Authors:** Kelechi Ugonna, Colin D. Bingle, Karen Plant, Kirsty Wilson, Mark L. Everard

**Affiliations:** 1 Department of Respiratory Medicine, Sheffield Children’s Hospital, Sheffield, United Kingdom; 2 Academic Unit of Respiratory Medicine, Dept. of Infection and Immunity University of Sheffield, Sheffield, United Kingdom; 3 School Of Paediatrics and Child Health, University of Western Australia, Princess Margaret Hospital, Subiaco, Western Australia; University ofTennessee Health Science Center, United States of America

## Abstract

We have previously shown that the respiratory syncytial virus [RSV] can productively infect monocyte derived dendritic cells [MoDC] and remain dormant within the same cells for prolonged periods. It is therefore possible that infected dendritic cells act as a reservoir within the airways of individuals between annual epidemics. In the present study we explored the possibility that sub-epithelial DCs can be infected with RSV from differentiated bronchial epithelium and that in turn RSV from DCs can infect the epithelium. A dual co-culture model was established in which a differentiated primary airway epithelium on an Air Liquid Interface (ALI) was cultured on a transwell insert and MoDCs were subsequently added to the basolateral membrane of the insert. Further experiments were undertaken using a triple co-culture model in which in which macrophages were added to the apical surface of the differentiated epithelium. A modified RSV [rr-RSV] expressing a red fluorescent protein marker of replication was used to infect either the MoDCs or the differentiated epithelium and infection of the reciprocal cell type was assessed using confocal microscopy. Our data shows that primary epithelium became infected when rr-RSV infected MoDCs were introduced onto the basal surface of the transwell insert. MoDCs located beneath the epithelium did not become infected with virus from infected epithelial cells in the dual co-culture model. However when macrophages were present on the apical surface of the primary epithelium infection of the basal MoDCs occurred. Our data suggests that RSV infected dendritic cells readily transmit infection to epithelial cells even when they are located beneath the basal layer. However macrophages appear to be necessary for the transmission of infection from epithelial cells to basal dendritic cells.

## Introduction

The respiratory syncytial virus [RSV] causes annual epidemics of respiratory disease affecting all age groups. Studies suggest that more than 60% of infants and 30% of the whole population experience a clinical illness due to RSV each winter [1]. The greatest impact of the virus is noted in infants with some 0.5–1% of all infants being admitted to hospital during the first winter after their birth [2], [3]. It is estimated that each year RSV is responsible for some 3.4 million hospitalizations and as many as 199,000 deaths worldwide [4]. It remains unclear why the virus is so successful and why it particularly affects very young infants at a time when passively acquired maternal antibodies are still at relatively high levels. Similarly the reason for the characteristic pattern of annual epidemics and the almost complete disappearance of the virus in the summer remains to be explained [5], [6]. The virus does not undergo significant antigenic shifts, as is the case for influenza, nor are there multiple circulating strains, as is the case for rhinovirus [7]. An alternative explanation for its success is that the virus is able to prevent the development of effective long-term memory responses thus permitting recurrent infections throughout life [8]. This would potentially contribute to poor herd immunity within the population and low levels of neutralizing antibodies amongst a significant proportion of pregnant mothers, placing a large proportion of infants at risk of infection during their first winter [9].

Key players in the development of effective immune responses to respiratory pathogens are the sub-epithelial dendritic cells [10], [11]. Previous work from our group has shown that RSV can infect monocyte derived dendritic cells (MoDCs) [12], [13] while a study involving infants hospitalized with RSV infections confirmed that dendritic cells numbers are significantly increased during and post infection and that HLA-DR+ve cells with the morphology of DCs contained RSV protein [14]. More recently we have shown that RSV is able to remain latent for prolonged periods within MoDC cells *in vitro* [15]. In these experiment we showed that re-activation of RSV replication within the MoDCs can be stimulated by exposing them to nitric oxide (NO) or a NO donor [16]. These studies would suggest that it is possible that RSV infection of DCs *in vivo* provides the virus with a niche in which it may remain dormant between epidemics.

In order to explore the possibility that virus may be passed between infected epithelium to sub-epithelial DCs and conversely from infected sub-epithelial DCs to an overlying differentiated epithelium we established dual *in-vitro* models. This involved establishing primary differentiated bronchial epithelial cell (pBEC) cultures on transwells insert and adding differentiated MoDC to the basal surface of the transwell. Further experiments were subsequently undertaken in which macrophages were added to the apical surface of the epithelium after infection of the epithelium in order to determine whether macrophages might play a role in the infection of MoDC. RSV has been shown to infect and replicate within macrophages [16]–[18]. Moreover, macrophages have been shown, in triple co-culture experiments, to play a direct role in the uptake of nanoparticles by sub-epithelial dendritic cells through direct cell to cell transfer of the particles [19]–[21].

## Methods

These in vitro studies were approved by the South Sheffield research Ethics Committee [08/H1310/92] and written informed consent was obtained from the healthy volunteers who donated blood from which the cells were prepared.

### Primary Bronchial Epithelial Cell (pBEC) Cultures

Cryopreserved human bronchial epithelial cells (HBEpC; Promocell, Heidelberg Germany) were cultured in Airway Epithelial Cell Growth Medium. (AEGM; Promocell, Heidelberg, Germany). Cells were obtained from two individual donors. They were seeded at 10×10^3^ cells/cm^2^ in T25 flasks and grown to confluency. Cells were subcultured with Trypsin-EDTA and aliquoted into new T25 or T75 flasks at 10–15×10^3^ cells per cm^2^. Cultures were received at Passage 2 and were used for experiments up to passages 4 or 5. At confluency, HBEpC were subcultured with Trypsin-EDTA and re-suspended in Air Liquid Interface (ALI) Media (Promocell, Heidelberg, Germany). Cultures were seeded at 0.8×10^6^ cells/ml. on the apical surface of 6.5 mm transwell ThinCerts with 3 μm pore size (FALCON; Becton Dickinson, NJ, USA) and placed in 24 well flat-bottomed plates (Corning Costar, High Wycombe, UK) with 0.75 mls of ALI media in the basal and upper compartments. Media in both compartments was changed daily until cell confluency was reached. Media was removed at confluency and cells were left exposed to air, to generate an ALI. The apical surface was washed daily with 200 μls of sterile PBS and basal media every 48 hours until the cells underwent mucociliary differentiation. This process took between 14 and 21 days. The differentiation of the cells was assessed by, immunofluorescence (IF) using staining for the goblet cell marker, MUC5AC and by western blotting for BPIFB1 (LPLUNC1), a protein that is only produced by fully differentiate ALI cell cultures [22], [23]. Cells were then ready to use for co-culture experiments and these were started when the cells had been at ALI for 21 days.

### Monocyte Isolation and Culture

Peripheral blood mononuclear cells (PBMCs) were isolated from freshly drawn venous blood using Histopaque gradient centrifugation. In brief, blood was obtained from healthy adult volunteers and anti-coagulated with heparin (Multiparin 1000 u/ml Heparin, CP Pharmaceuticals Ltd Wrexham) at a concentration of 20 units/ml of blood. The blood was diluted to 50% with Monocyte isolation buffer (MIB), consisting of Endotoxin free PBS without Calcium/Magnesium, 2% FCS, 0.002% EDTA and layered at 5∶2 ratio on to Histopaque 1077 solution. (Sigma Chemical Co, Poole, UK). The gradient was then centrifuged at 400 g for 40 minutes at room temperature with no brake. The interphase of mononuclear cells was removed and re-suspended in MIB to desired concentration. The cell solution was centrifuged at 300 g for 10 minutes. The supernatant was discarded and cells were re-suspended in 20 mls of MIB and counted using a haemocytometer.

Enrichment of the monocyte population was achieved by the EASYSEP negative immunomagnetic selection method (STEMCELL Technology, Genoble, France). The PBMC cell solution from above was centrifuged at 300 g for 10 minutes and supernatant was discarded to remove the remaining histopaque and plasma. The cell pellet was resuspended in MIB at a cell density of 5×10^7^ cells/ml. EasySep Human Monocyte Negative Enrichment Cocktail was added at 50 μl/ml to the cell solution, mixed well and incubated at 4°C for 10 minutes. EasySep Magnetic microparticles were then added at 50 μl/ml, mixed well and incubated at 4°C for 5 minutes.

The cell suspension was then placed in an EasySep magnet and incubated for 2.5 minutes at room temperature. The remaining cells were re-suspended in MIB and solution replaced in the EasySep magnet for a further 2.5 minutes.

### Monocyte Derived Dendritic Cell (MoDC) Culture

MoDCs were isolated as previously described [12], [13], [15]. Briefly an enriched monocyte suspension was centrifuged at 300 g for 10 minutes at room temperature and the cells were re-suspended at a density of 1×10^6^ cells/ml in serum free X-Vivo-20 (BioWhittaker, Wokingham, Berkshire, UK). The cell suspension was aliquoted at 0.5 mls per well into 24 well flat bottomed plates (Corning Costar, High Wycombe, UK). Cells were supplemented with 40 ng/ml GM-CSF (Biosource International, Paisley, UK) and 20 ng/ml IL-4 (Biosource International, Paisley, UK) and were cultured at 37°C in a 5% CO2/air mix Sanyo humidified incubator for 5–7 days to allow for maturation to dendritic cells. Cells were characterized by the use of surface markers specific for DCs (CD83 CD86) and markers specific for co-presentation (CD40 and HLA Class II). Cultures were given 50% fresh media containing cytokines twice weekly. Half of the MoDCs were infected with red fluorescent rr-RSV at 5×10^5^ pfu/ml (MOI of 0.5). All the cells were cultured under standard conditions for another 5–7 days at which point they were used in co-culture experiments.

### Monocyte Derived Macrophage (MDMs) Culture

Enriched monocytes were centrifuged at 300 g for 10 minutes at room temperature and cells re-suspended at a density of 1×10^6^ cells/ml in RPMI 1640 media containing L-glutamine and NaHCO3 (Sigma, Poole, UK) supplemented with 10% FBS (Gibco, Paisley, UK). The cell suspension was aliquoted at 0.5 mls per well in 24 well flat-bottomed plates. All non-adherent cells were removed on day one and cultures were given 50% fresh media twice weekly. Cells were cultured at 37°C in a 5% CO2/air mix Sanyo humidified incubator for 14 days for differentiation to MDMs using established protocols developed in our unit [24].

### Virology

An A2 RSV virus carrying the gene for Red Fluorescent Protein (RFP), designated rr-RSV was used in these experiments. This virus, which generates RFP only during active replication, was kindly obtained from Dr Mark Peebles (Nationwide Children’s Hospital, Columbus). The preparation of virus has previously been described [25].

For viral propagation, HeLa cells were grown to 80% confluence in T75 flasks, the growth media was removed and 5 mls of a 1∶2500 dilution of RSV was added. Following incubation for 2 hours, with the media being redistributed by tipping every 20 minutes, 10 mls of DMEM supplemented with 2% FCS, 2 mM L-Glutamine and 1% Penicillin and Streptomycin was added. The cells were left for 2–3 days under normal culture conditions until significant fluorescence was observed in the cells and they were then harvested by scraping. Cells were broken up to release intracellular virus by vigorous pipetting, passing through a 21 gauge needle a number of times and rapid freeze: thaw using liquid nitrogen. Any intact cells were removed by centrifugation at 250 g for 5 minutes and the supernatant was snap-frozen in liquid nitrogen and stored at −80°C. In order to obtain purified virus the preparation was centrifuged through a Vivaspin-20 ultra-filtration tube (Vivaspin, Saertorius, Goettingen, Germany) to remove low molecular weight contaminating protein as previously described [26]. This simple method does not affect the infectivity of the virus. The purified virus was snap-frozen in liquid nitrogen and stored at −80°C together with samples of filtrate which were used as a control. Virus titre was determined by a standard plaque assay.

### Generation of Transwell Co-cultures

**Figure 1:**
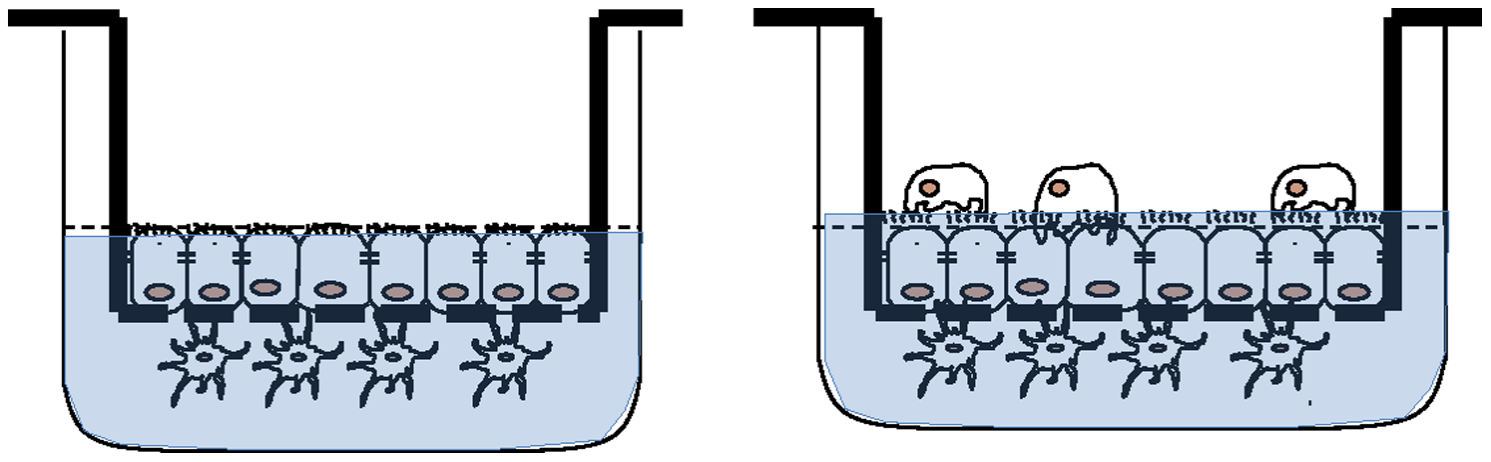
Schematic representation of dual and triple co-cultures. pBEC = primary bronchial epithelial cell culture, MoDC = monocyte derived dendritic cells, MDM = monocyte derived macrophages.

For these experiments dual and triple trans-well co-cultures were established. For dual co-cultures a pBEC was established on a transwell insert and subsequently a MoDC culture was established on the contralateral surface of the insert. For triple cultures MDMs were subsequently added to the apical surface of the pBEC following successful establishment of the duel co-culture. This is represented schematically in Figure 1 and described in more detail below.

This technique was based on a published method developed using lung cell lines [19]–[21]. pBECs differentiated at the ALI were used on the apical surface of the co-culture model. Epithelial cells were differentiated on the apical surface of BD Falcon 24 well cell-culture transwell inserts (surface area 0.3 cm^2^, pores of 3 microns, BD Biosciences, Claix France) as described above.

When the cells were confluent the inserts were turned upside down and placed in a well from a 6 well culture dish. (Corning,NY, USA). A cell scraper was used to gently abrade any epithelial cell material that may have grown through the membrane. The membrane was washed once with RPMI 1640 medium. 0.5 mls of infected or uninfected MoDC cell suspension (5×10^5^ cells) was added to the basolateral membrane of insets. The culture dish was covered and cultured for 2 hours. Inserts were then reverted and placed back in 24 well plates and 0.25 mls of standard MoDC media was added to the bottom of the well.

For the triple co-cultures, 0.5 mls of MDM cell suspension [approx. 1×10^6^ cells/ml] was added to the apical surface of the pBECs. Cells were allowed to attach for 2 hours and non-adherent cells were washed away with 0.5 mls of PBS.

Co cultures were infected with rr-RSV at 1×10^6^ pfu/ml (MOI of 1). For experiments involving direct infection of the epithelium the virus was added to the apical surface of the pBECs. The infection was allowed to proceed for 2 hours, with gentle agitation every 20 minutes and then the cells were washed with 0.5 mls of PBS three times. For indirect infection the MoDCs were exposed to virus for 2 hours prior to their addition to the basal surface of the cell insert.

The experiments with the dual and triple co-culture are outlined in Table 1. All the experiments were repeated 6 times with 3 sets of each co-culture. Three different donors were used to provide MoDCs and MDMs. In experiments involving both dendritic cells and macrophages these cells were obtained from the same donor.

**Table 1:** Overview of experiments undertaken.

Experiment	Description	Cells primarily infected with RSV
1	Dual culture	Mock
2	Dual culture	pBEC
3	Dual culture	MoDC
4	Triple culture	pBEC

pBEC = primary bronchial epithelial cell culture, MoDC = monocyte derived dendritic cells.

### Microscopy

Microscopy and photography was performed on live cells, using a LEICA time phase microscope, just prior to preparation for cells for Flow cytometry. IF localisation of MUC5AC was performed on acetone/methanol fixed filters as previously described [23].

### Westen Blotting

5 μl aliquots of apical washes from the ALI pBECs were denatured, resolved on 12% SDS-PAGE gels and western blotted using specific antibodies against human SPLUNC1/BPIFA1 (1∶500 dilution)^23^. Detection was performed using ECL and x-ray film (Amersham) following incubation with HRP conjugated secondary antibody (1∶2000 dilution).

### Preparation of Cells for Flow Cytometry

#### pBEC

Cells on the apical surface of the transwells were washed with 0.2 mls of PBS. 0.2 ml of trypsin was added to each well and incubated at 37°C for 5 minutes or until the cells dissociated. 0.3 ml of RPMI media was added to each well to quench the trypsin reaction and the cells were placed in a 1 ml ependorf tube. Cell dissociation was confirmed by light microscopy. Cells were spun at 2500 rpm for 5 minutes, the supernatant was discarded and the cells were re-suspended in 0.5 mls of FACS buffer (0.1% Bovine Serum Albumin, BSA, in PBS) and placed in a FACS tube. The cells were spun at 2500 rpm for 5 mins, the supernatant was discarded and the cells were finally re-suspended in 0.4 ml FACS buffer. They were placed on ice, ready for flow cytometry.

#### MoDCs

The transwells were turned upside down and cells were dissociated from the basolateral surface by gentle scraping with a cell scraper. 0.2 ml of PBS was placed on the basolateral surface and cells were removed and prepared for flow cytometry as above.

Flow cytometry was performed with a FACsCalibur machine, in the University of Sheffield’s core research facility.

### Statistical Analysis

The mock exposed cells acted as negative controls for all studies. Intensity of fluorescence for each population was calculated using geometric mean and collated using FlowJo software. In these studies increased red fluorescence is assumed to reflect an increased proportion of RSV infected cells in the cultures. Data was analysed using a one-way ANOVA statistical test using the statistical program Prism.

## Results

**Figure 2:**
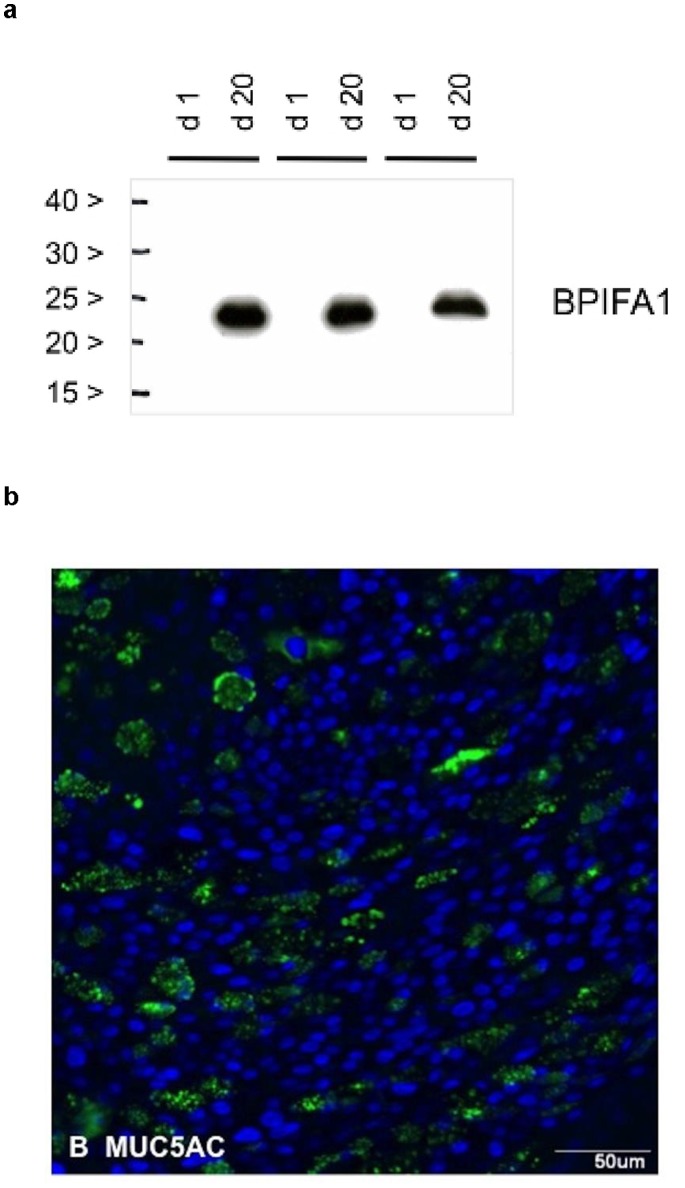
Confirmation of differentiation of the ALI cultures. **A.** Western blotting of apical cell washes were used to show induction of SPLUNC1/BPIFA1 in primary cells by day 20 compared to day 0 of ALI culture. **B.** A 20 day ALI culture was stained for MUC5AC as outlined in the methods section. The positive green staining shows the presence of goblet cells within the cell layer after differentiation.

The pBECs cultured at the ALI were shown to produce markers of differentiation including BPIFA1 by western blotting of apical washes (Figure 2a) and MUC5AC shown by IF microscopy (Figure 2b) thus confirming that the primary cells had effectively differentiated into a mucociliary phenotype representative of human airway epithelium.

### Direct and Indirect Infection of Differentiated pBECs

**Figure 3:**
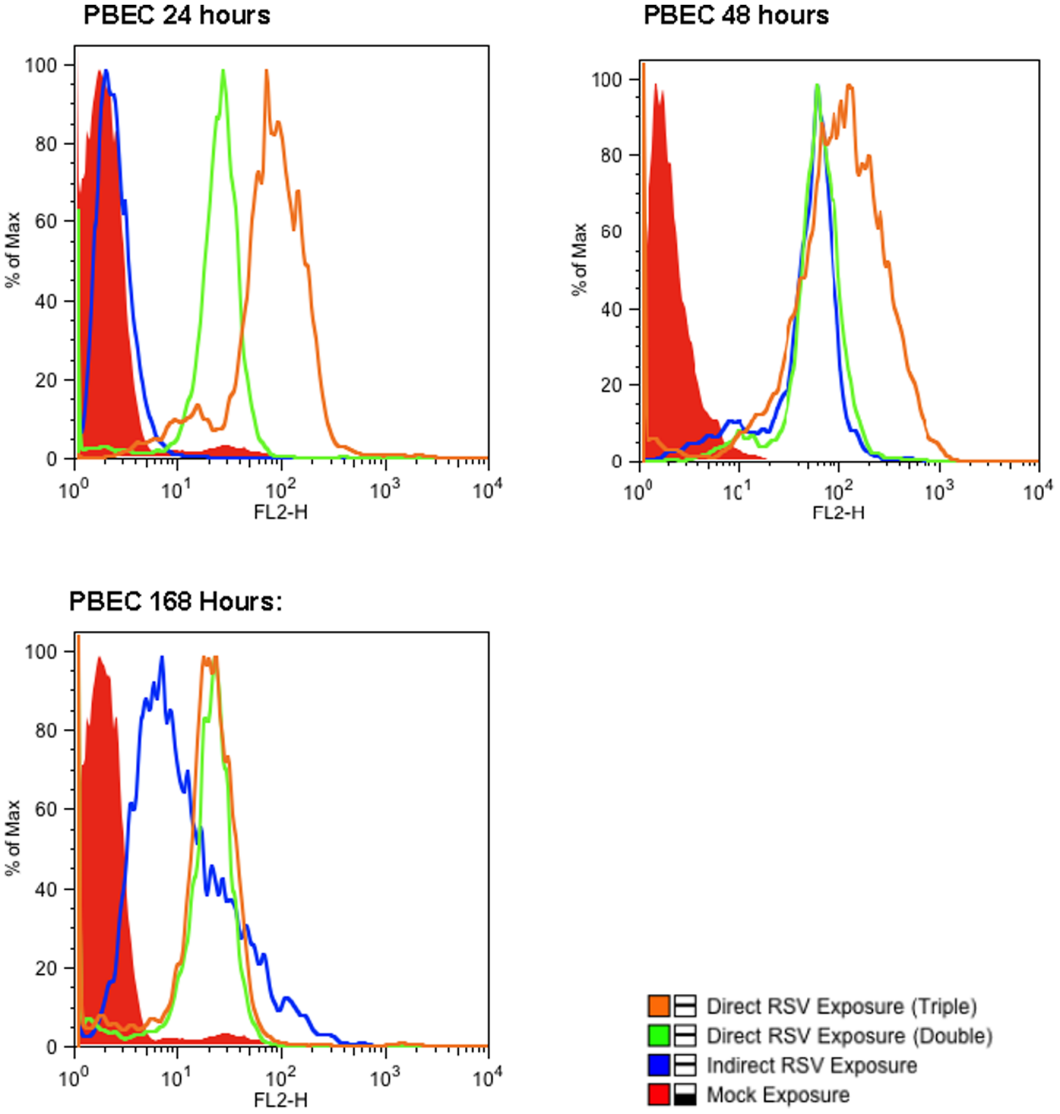
Active RSV infection occurs in both directly and indirectly infected pBECs. Cell infections and FACS analysis were performed as outlined in the methods section. Direct exposure means pBECs were directly infected with RSV, whereas indirect exposure means that the RSV infected MoDCs were the source of the virus. The image shows a representative example of flow cytometry results for pBECs from a co-culture experiment.

**Figure 4:**
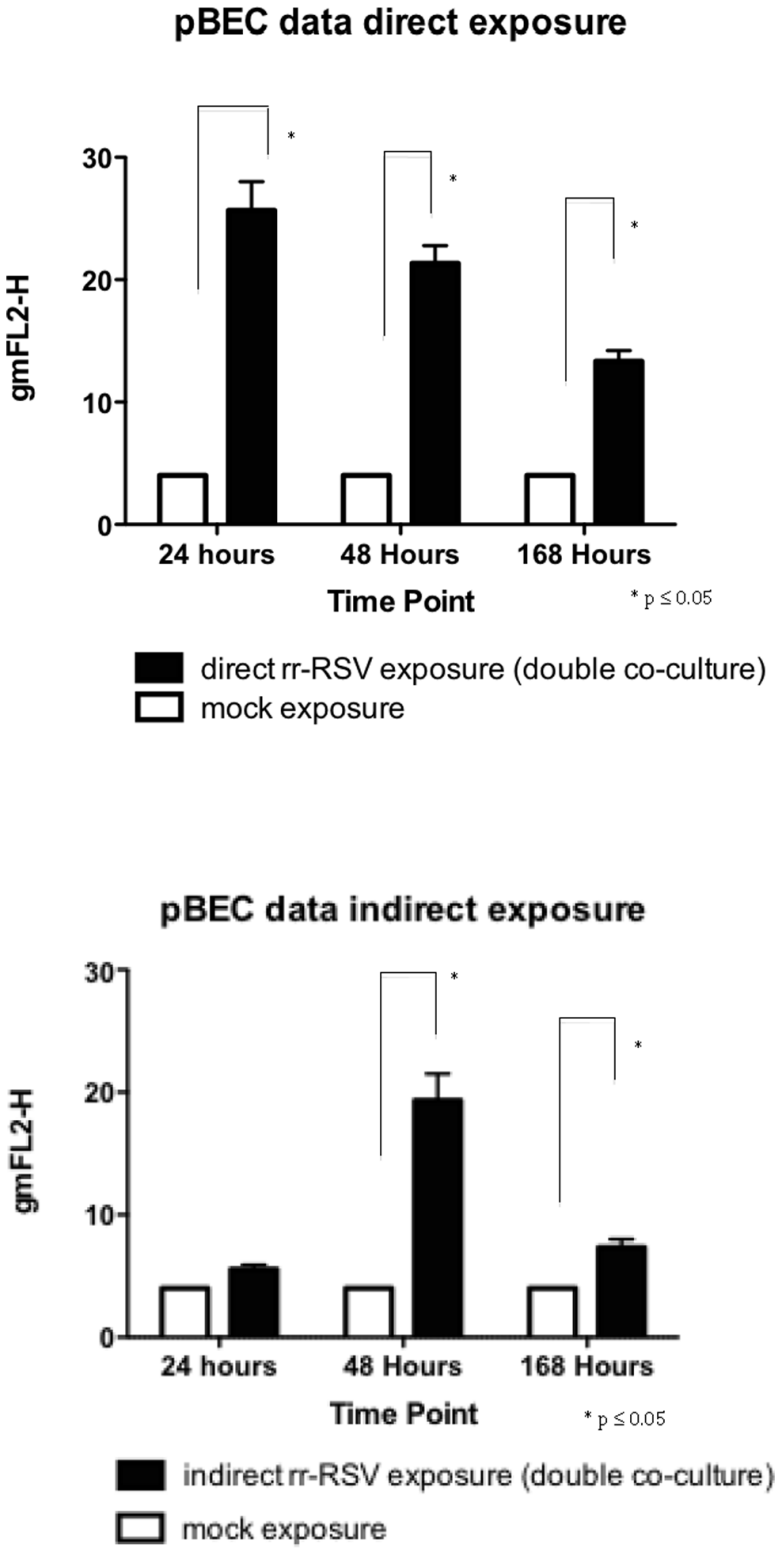
Collated fluorescence data from directly and indirect exposure studies. Cell infections and FACS analysis were performed as outlined in the methods section. The data represents 6 experiments performed in pBECs with standard error of the mean shown by the error bars.

**Figure 5:**
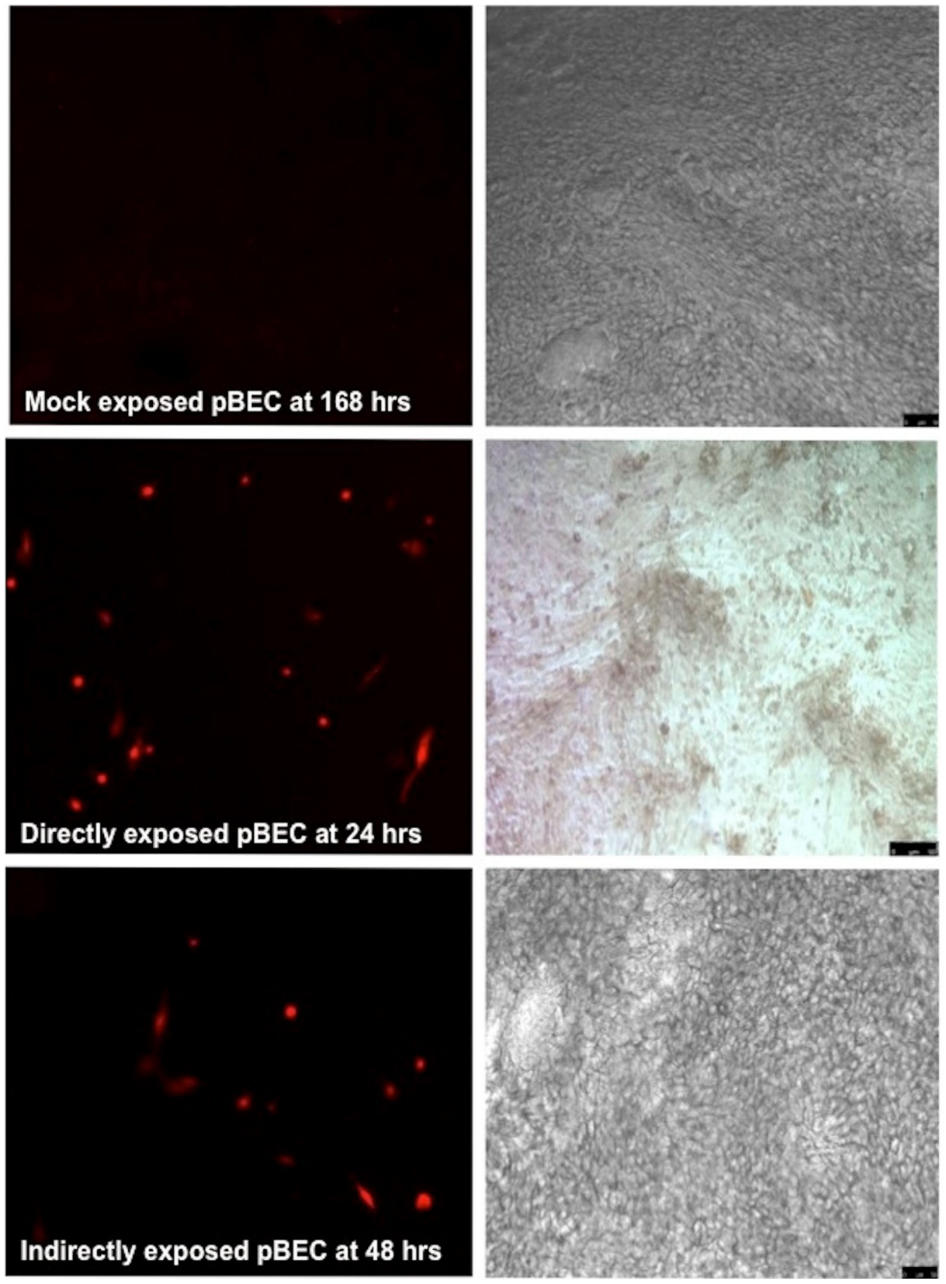
Active RSV replication is seen in both directly and indirectly infected pBEC cultures. Cell infections and IF microscopy were performed as outlined in the methods section. The images show active RSV replication (as indicated by red fluorescence) in pBEC images under fluorescence microscopy at the indicated time alongside corresponding phase contrast images of the pBEC cultures.

Infection and replication of RSV was evident in the directly exposed pBECs at 24 hours post inoculation as shown by robust detection of red florescence. This was still present at 168 hours of culture (Figure 3). The pBECs also became infected with RSV following the addition of infected MODCs to the basal surface of the transwell insert (Figure 3) though the intensity of staining fluorescence was reduced. Quantitation of data from 6 individual experiments confirmed that RSV readily infected the primary epithelial cultures when virus was introduced directly onto the apical surface and when exposed to MODCs introduced below the insert. Compared with the control, mock infected, pBECs, there was a significant increase in fluorescence in the directly exposed pBECs at 24, 48 and 168 hours (Figure 4). For confirmation of this observation we used IF microscopy and corresponding phase contrast analysis, to show active RSV infection (as judged by red florescence) within the epithelial cell layer (Figure 5). These phase contrast images also appeared to show that the differentiated ALI cultures retained their viability throughout the experimental periods.

### Infection of MoDCs with RSV in the Sub-epithelial Space

**Figure 6:**
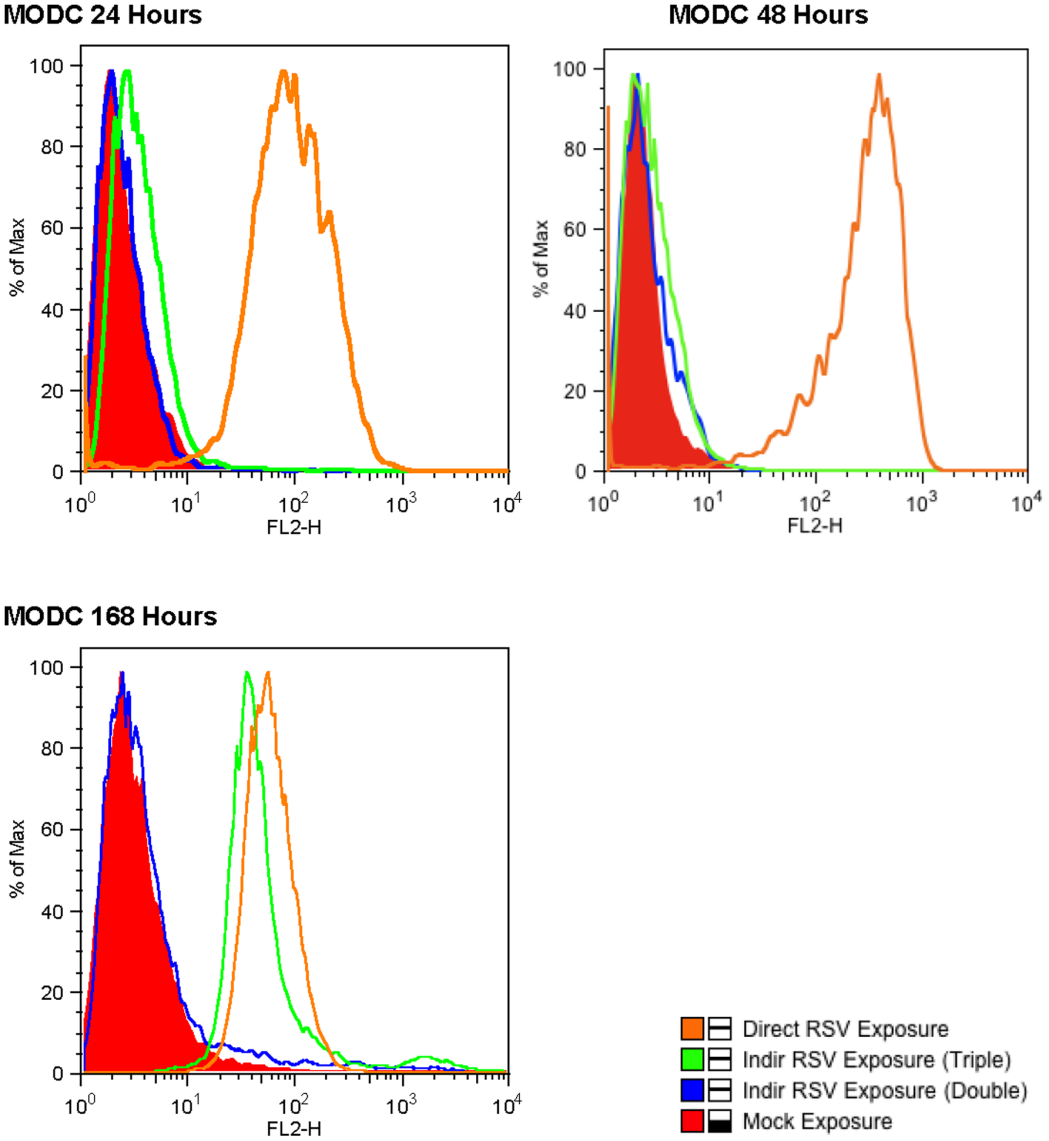
Active RSV replication is seen in both direct and indirectly infected MoDCs. Cell infections and FACS analysis were performed as outlined in the methods section. Direct exposure means MoDCs were directly infected with RSV, whereas indirect exposure means that the RSV infected pBECs were the source of the virus. This is shown in an example of flow cytometry results of MoDCs on co-culture from a single experiment.

Direct exposure of MoDCs to RSV resulted in infection of these cells as determined by flow cytometry (Figure 6). As was the case with direct exposure of the of the pBEC to RSV, there was clear evidence of MoDC infection at all time points.

In contrast we were unable to identify evidence of MoDCs located on the basal layer of the transwell inserts becoming infected at any time point after infection of the pBEC with RSV despite vigorous replication within the differentiated epithelium (Figure 6). However, the addition of macrophages to the epithelial surface following infection of the epithelium resulted in clear evidence of RSV infection of the MoDCs at 168 hours after infection of the epithelium (Figure 6).

**Figure 7:**
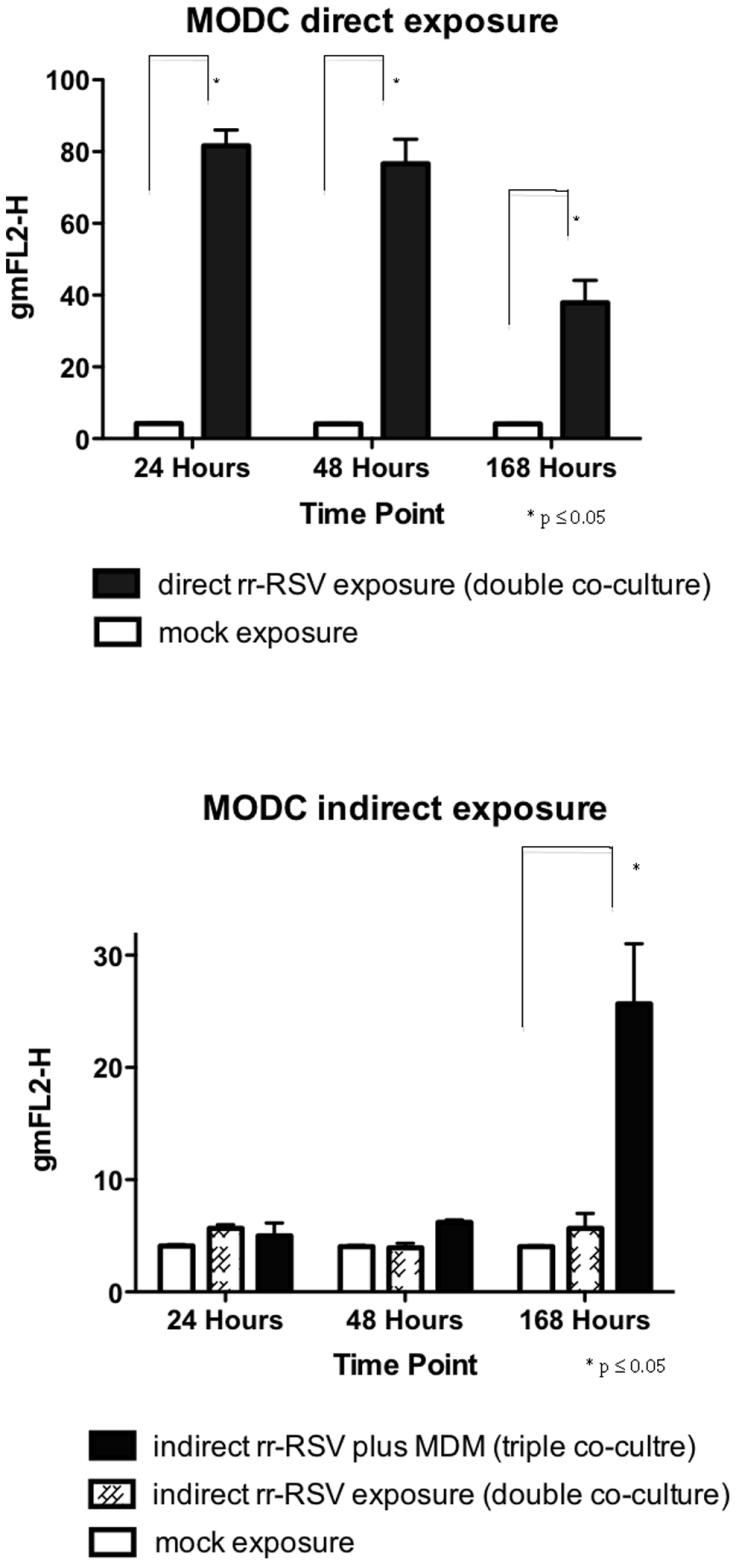
Collated fluorescence data from direct and indirect exposure studies. Cell infections and FACS analysis were performed as outlined in the methods section. The data shown represent data from 6 experiments performed in MoDCs with standard error of the mean shown by the error bars.

**Figure 8:**
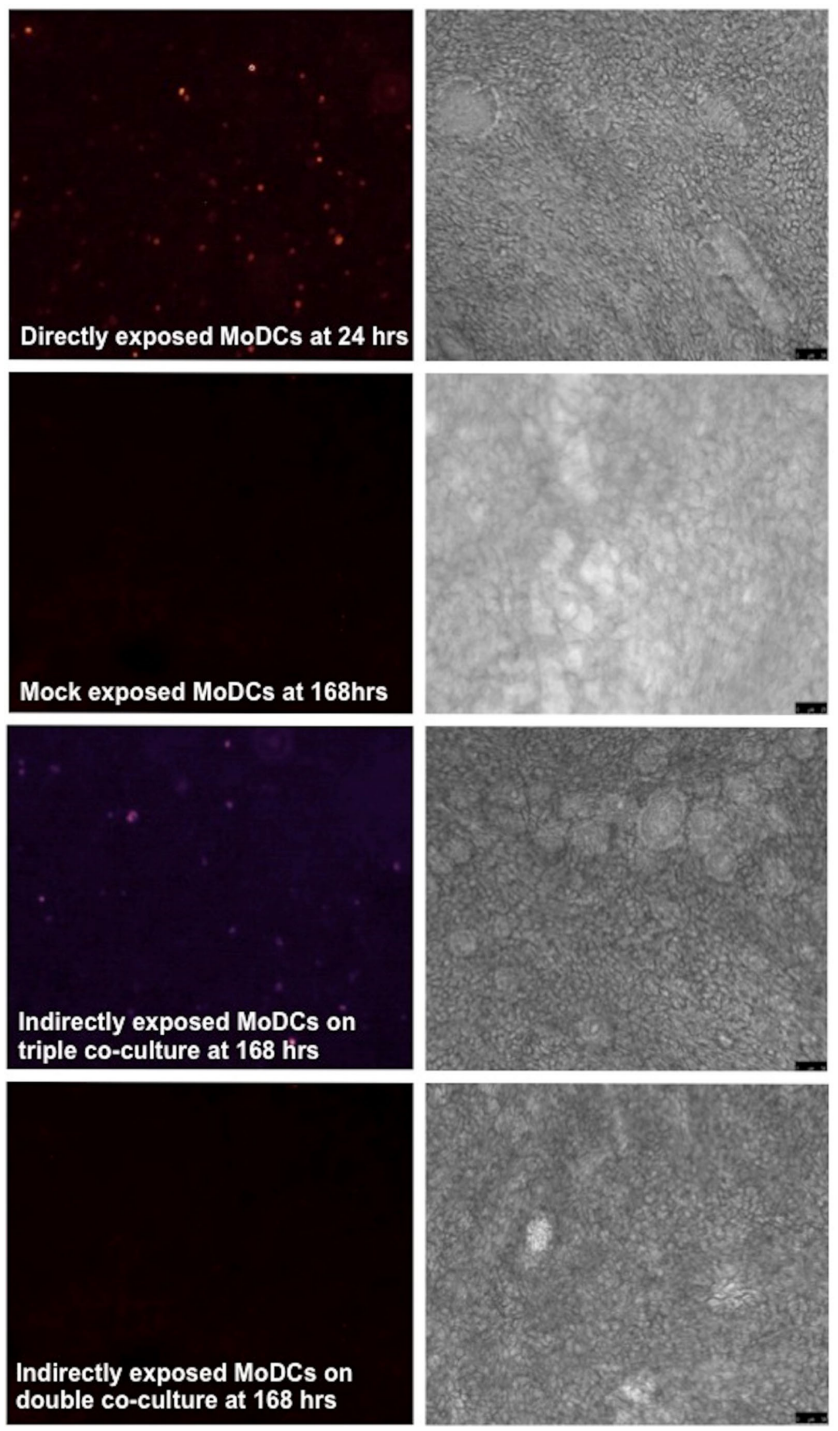
Active RSV replication is seen in directly infected MoDC cultures by 24 hours. Cell infections and IF microscopy was performed as outlined in the methods section. The images show active RSV replication (as indicated by red fluorescence), alongside phase contrast images of the cultures.

Quantitation of infection data from 6 experiments confirmed that MoDCs directly exposed to RSV showed evidence of productive infection at all three time points (Figure 7), with levels at 24 and 48 hours being higher than that seen at 168 hours. In the absence of apical macrophages there was no fluorescence detectable by flow cytometry, in the MoDCs indirectly exposed to rr-RSV through the infected pBECs at any time point. However in the presence of MDMs there was a significant increase in fluorescence in the indirectly exposed MoDCs at 168 hours (Figure 7). Representative IF images and corresponding phase contrast images from these experiments are shown in Figure 8. Again these microscope images of the epithelium did not indicate that the presence of macrophages had an effect on the integrity of the epithelium at 168 hrs (Figure 8) and no leakage of basolateral fluid was detected at the apical surface of the cultures, suggesting that the epithelial layer was functionally intact.

## Discussion

Our data confirm previous studies that show that RSV readily infects differentiated airway epithelial cell cultures. More significantly, our data also suggests that RSV replicating in dendritic cells lying beneath a continuous differentiated primary bronchial epithelium can directly infect the airway epithelium. However, in the same co-culture model, sub-epithelial dendritic cells do not themselves become infected with RSV following primary infection of the epithelium unless macrophages are present on the apical surface of the epithelium. We believe that these data support the suggestion that RSV infection of the bronchial epithelium might infect sub-epithelial dendritic cells and in turn these cells may infect overlying epithelial cells.

It has previously been established that infection of bronchial epithelium with RSV is polarized, with the virus only able to infect the cells via their apical surface [27]. This suggests that the infected MoDCs in our cultures may have released virus onto the luminal surface. Since we did not identify any evidence of the MoDCs migrating through the membrane and the epithelium appeared confluent and patent, across all the inserts, it is possible that the virus is released from ‘snorkeling’ dendrites that reach through to the surface of the epithelium from the sub-epithelial compartment [28], [29]. Intriguingly sub-epithelial dendritic cells did not become infected in the presence of infected epithelium unless macrophages were present on the apical epithelial surface. Dendritic cells are known to be very important in processing respiratory pathogens and traditionally have been assumed to take up pathogens directly through ‘snorkeling’ dendrites. In our experiments infection of the epithelium is likely to have activated the DCs through the release of a number of cytokines that have previously been shown to be induced following RSV infections and hence they were likely to be primed to take up virons making the lack of uptake more surprising.

Further work is required to elucidate the mechanisms leading to infection of the MoDCs in the triple co-culture model. Previous studies involving primary differentiated epithelial cultures have confirmed that RSV causes little in the way of cytopathy despite evidence of on-going replication even 3 months after the initial infection [27]. However, it is possible that the integrity of the epithelium was compromised in the presence of macrophages thus permitting the DCs to access the virus. Our phase contrast microscopy images did not appear to show any significant epithelial damage in the presence of macrophages as compared with the dual co-culture pBECs. Cultures were still confluent at 168 hrs and we did not observe any leakage of media in to the apical compartment of the transwell, suggesting that the culture retained its integrity.

A possible alternative explanation is that macrophages are necessary to for virus to infect the DCs. In recent studies using inert nano-particulates in a similar triple co-culture model, it was shown that macrophages on the luminal surface of the airways contribute to the process of translocation of nano-particles across the epithelium by taking them up at the epithelial surface and then passing them directly to sub-epithelial DCs [19]–[21] which in turn can also pass particulates on to other dendritic cells [30]. In these studies imaging demonstrated the direct interaction of ‘snorkeling’ dendrites and the macrophages. The reported rate of transfer of nanoparticles from macrophages to DCs was highly variable depending on factors such as particle size, charge and dose. These studies utilized a simple monolayer cell line in contrast to the complex differentiated epithelium we have used in these experiments.

We suggest that a similar to the situation may occur with viruses such as RSV. As noted above it is known that RSV infects and is released from the apical surface of epithelial cell [27]. Previous *in vitro* work has shown that the virus is taken up by macrophages and indeed can productively infect the [16]–[18]. It is possible that uptake by macrophages is a necessary step for sub-epithelial dendritic cells to acquire the virus similar to that described for particulates though the mechanism needs to be explored. The lack of infection of indirectly exposed MoDCs at the 48 hr time point is likely to be due to a number of factors. In order to be transferred via macrophages there is a need for RSV first to replicate in the epithelium prior to being taken up by, and potentially replicating in, macrophages. The use of a differentiated epithelium may also have influenced the timing of infection of the sub-epithelial MoDCs with these cultures forming much more efficient tight junctions than the monolayers used in the nanoparticle experiments. Unfortunately there were no intermediate time points between 48 and 168 hours and hence we are unable to comment on the dynamics of MoDC infection between these two time points.

It is of interest that the presence of MDMs appeared to promote viral replication when virus was added directly to the apical surface of the pBECs. It is known that the virus replicates in macrophage [16]–[18] and hence this may account for the apparent increase.

In summary these experiments suggest that RSV from infected sub-epithelial DCs can infect a differentiated bronchial epithelium. It would appear that for the infected epithelium to infect subepithelial DCs, surface macrophages need to be present.
